# Asthma in the Brazilian Unified Health Care System: an epidemiological analysis from 2008 to 2021

**DOI:** 10.36416/1806-3756/e20230364

**Published:** 2024-05-08

**Authors:** David Halen Araújo Pinheiro, João Victor Hermógenes de Souza, Alberto Fernando Oliveira Justo, Regina Maria Carvalho-Pinto, Fabiano Francisco de Lima, Celso R F Carvalho

**Affiliations:** 1. Departamento de Fisioterapia, Faculdade de Medicina, Universidade de São Paulo - USP - São Paulo (SP) Brasil.; 2. Laboratório de Fisiopatologia do Envelhecimento, Departamento de Clínica Médica, Universidade de São Paulo - USP - São Paulo (SP) Brasil.; 3. Divisão de Pneumologia, Instituto do Coração - InCor - Hospital das Clinicas Faculdade de Medicina, Universidade de São Paulo, São Paulo (SP) Brasil.

**Keywords:** Asthma/mortality, Asthma/epidemiology, Hospitalization, National health programs

## Abstract

**Objective:**

To analyze the number of hospitalizations, the length of hospital stay, and mortality due to asthma, as well as the costs to the Unified Health Care System in Brazil between 2008 and 2021.

**Methods:**

This was a cross-sectional epidemiological study using data from the Information Technology Department of the Brazilian Unified Health Care System. Proportional hospitalization and death rates were estimated per 100,000 population by age, microregion, and year.

**Results:**

The number of hospitalizations and deaths due to asthma decreased from 2008 to 2021 (205,392 vs. 55,009 and 822 vs. 327, respectively). In addition, a between-sex difference was observed in asthma-related hospitalizations in 2008, and more men were hospitalized in 2021 (51.8%). Asthma mortality rates were similar for both sexes (50.0% each) in 2008, and a slight increase was observed in women’s deaths in 2021 (52.9%). Even so, approximately one death/day and more than 55,000 hospitalizations were observed yearly, with a mean length of hospital stay of three days. Additionally, the Southeast region allocated more financial resources to asthma-related hospitalizations.

**Conclusions:**

Our results showed that the number of deaths and hospitalizations due to asthma substantially declined during the study period.

## INTRODUCTION

Asthma is the second most common chronic respiratory disease and affects around approximately 300 million people worldwide.^1^ Moreover, it is estimated that 20 million people are affected by this disease in Brazil.^2^

A recent study showed that the prevalence of asthma in Brazil is 4.6% and 12.1% in adults and children, respectively.^3^ Although most cases are manageable with pharmacological treatment, such as inhaled corticosteroids (ICSs) and long-acting β_2_ agonists (LABAs), up to 10% of patients have severe asthma and require further treatment.^4^ Severe asthma is associated with increased morbidity and mortality and negatively impacts a patient’s psychological condition and socioeconomic status.^5^

The costs per patient with severe asthma can be up to ten times greater than those per patient with the mild form of the disease,^6^ and severe asthma accounts for more than 60% of health expenses related to asthma. The costs of asthma can be high when the disease is not controlled.^7^ In Brazil, 71.5% of the population depends on the public health care system, which provides services at all levels of care.^8^ Considering the number of people with asthma in the country, this disease generates high costs for the government through health services.

Throughout the current century, there have been changes in Brazilian health policies. Some Brazilian cities have offered ICSs free of charge to their inhabitants since 2003^9^; however, in 2009, the Unified Health Care System (UHCS) included ICSs for people with asthma.^10^ This represented higher expenses associated with medication but potentially lower expenses associated with hospitalization.

The WHO calls for “better surveillance to map the magnitude of chronic respiratory diseases and analyze their determinants and monitor future trends.”^11^ Although the *Departamento de Informática do Sistema Único de Saúde* (DATASUS, Information Technology Department of the Brazilian Unified Health Care System) continuously updates the data on asthma, it is necessary to organize and analyze these data to draw conclusions and identify room for improvement in public policies. To the best of our knowledge, the latest study systematically assessed data related to the economic impact of asthma nationally through 2013.^10^

Assessing the regional impacts of asthma is important, particularly in countries with a vast area such as Brazil, characterized by substantial variations among its macroregions. A study showed a downward trend in asthma mortality in the early 2000s in Brazil, although regional disparities persisted.^12^ The Northeast region was the sole area where mortality rates did not decrease, likely influenced by variations in health care access and climatic conditions.^12^ ^-^ ^14^

Considering the reported advances in asthma management in the last decade, it is important to update the data regarding this disease. The Global Asthma Report,^15^ prepared by the WHO, reinforces the need for continuous monitoring of asthma to investigate trends in this disease. The present study aimed to update the data referring to hospitalization, mortality, and expenses associated with asthma by the Brazilian UHCS by examining regional differences and relevant changes according to changes in public policies.

## METHODS

This epidemiological study, conducted in October 2022, assessed hospitalization and mortality rates due to asthma and status asthmaticus as defined by the International Classification of Diseases, 10th revision (ICD-10) according to the sex and the subject’s geographic macroregion within Brazil. The inclusion criteria were defined as living in Brazil between January of 2008 and December of 2021.

The financial costs, hospitalization numbers, length of hospital stay, and mortality data were extracted from the DATASUS Ministry of Health of Brazil website.^16^ The data available in DATASUS are part of the universal accessibility policy of the Brazilian UHCS and include the Hospital Information System and Mortality Information System (hospitalization and mortality data, respectively), which are composed of registers collected through city hall health departments. The data collection methodology did not change during the study period. Therefore, the individuals whose information was extracted were deidentified. This study did not require approval from a research ethics committee.

The DATASUS information includes the basic and associated causes based on the ICD-10 codes, in which the codes ‘J45’ and ‘J46’ represent asthma and status asthmaticus, respectively. The demographic data were collected from the *Instituto Brasileiro de Geografia e Estatística* (IBGE, Brazilian Institute of Geography and Statistics) website.^17^ The IBGE runs a census every 10 years to verify the Brazilian population profile by collecting a number of variables from every household in the country; the sociodemographic profiles in the years between censuses are estimated through projections.

### Data analysis

For the proportional hospitalization and death rates, we used the number of hospital admissions or deaths by sex, region, or calendar year as the numerator and the respective number of people in this range as the denominator, as shown in the following equations^18^:

$$\;Hospitalization\;\frac{\;age\;}{\frac{\;region\;}{\;year\;}}=\frac{\;number\;of\;hospitalizations\;}{\;total\;population\;in\;the\;range\;}\times 10^{5}$$

and

$$\;Mortality\;\frac{\;age\;}{\frac{\;region\;}{\;year\;}}=\frac{\;number\;of\;deaths\;}{\;total\;population\;in\;the\;range\;}\times 10^{5}$$

The monetary restatement was calculated with the *Índice de Preços ao Consumidor* (IPCA, National Consumer Price Index), one of Brazil’s most traditional and important inflation indices. The following formula was used to calculate the monetary restatement during the study period:

$$\;Corrected\;\cos t\;=\frac{\;Average\;\cos t\;}{1000}\times \;Year\;IPCA\;tax\;$$

Data analyses were conducted using Prism, version 8.0 (GraphPad Software, San Diego, CA, USA).

## RESULTS

From 2008 to 2021, Brazil had more than 8,000 deaths and more than 1.7 million hospitalizations due to asthma. The Northeast region had the highest absolute number of deaths and hospitalizations among men and women, and the Central-West region had the lowest during this period. The Southeast was the only region where more men than women were hospitalized, and in the North and Central-West regions, there were more deaths among men than among women, as opposed to the other three regions. Table 1 and Table 2 show the total number of hospitalizations and deaths per year from 2008 to 2021 and those numbers by region and sex.

**Table 1:** Frequency of hospitalization and deaths from asthma and status asthmaticus per year.^a^

Year	Hospitalization				Death			
	Men		Women		Men		Women	
	n	Per 100,000	n	Per 100,000	n	Per 100,000	n	Per 100,000
2008	101,993	107.6	103,399	106.9	411	0.43	411	0.42
2009	99,950	104.4	103,247	105.6	411	0.43	448	0.46
2010	95,576	98.8	97,621	98.8	442	0.46	447	0.45
2011	86,951	89.1	91,271	91.5	394	0.40	378	0.38
2012	73,110	74.2	75,092	74.5	346	0.35	407	0.40
2013	65,822	66.3	68,500	67.4	314	0.32	383	0.38
2014	56,740	56.6	59,659	58.1	254	0.25	333	0.32
2015	56,285	55.8	57,445	55.5	243	0.24	300	0.29
2016	47,125	46.3	47,893	45.9	256	0.25	303	0.29
2017	46,587	45.5	46,590	44.3	197	0.19	288	0.27
2018	43,495	42.	43,601	41.1	189	0.18	242	0.23
2019	39,712	38.2	40,235	37.7	204	0.20	241	0.23
2020	23,787	22.8	24,175	22.5	150	0.14	178	0.16
2021	28,493	27.1	26,516	24.5	154	0.15	173	0.16
Total	865,626		885.244		3,965		4.532	

a Hospitalizations and deaths normalized per 100,000 population per year.

**Table 2:** Frequency of hospitalization and deaths from asthma and status asthmaticus per region.^a^

Region	Hospitalization				Death			
	Men		Women		Men			Women
	n	Per 100,000	n	Per 100,000	n	Per 100,000	n	Per 100,000
North	92,534	75.3	95,461	80.0	209	0.17	193	0.16
Northeast	374,155	96.9	385,193	96.0	1,430	0.37	1,702	0.42
Southeast	212,393	36.1	202,834	33.5	1,375	0.23	1,601	0.26
South	124,374	61.8	138,274	67.2	597	0.30	719	0.35
Central-West	62,170	58.3	63,482	59.0	354	0.33	317	0.29
Total	865,626		885,244		3,965		4,532	

a Hospitalizations and deaths normalized per 100,000 population per region.

**Figure 1:**
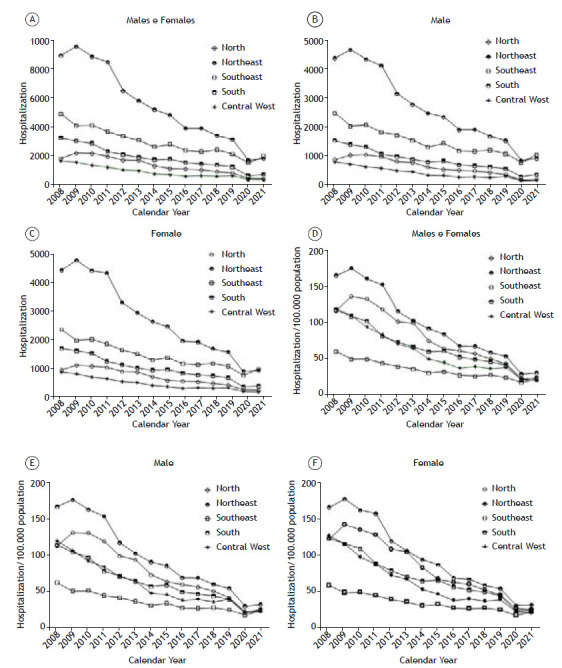
Number of hospitalizations due to asthma per year by Brazilian macroregions. In A, hospitalizations in total population. In B, hospitalizations in the male population. In C, hospitalizations in the female population. In D, hospitalizations per 100,000 population. In E, hospitalizations per 100,000 males. In F, hospitalizations per 100,000 females.

During the study period, there was a decrease in hospitalizations in the general population among men and women (Figure 1A). When the respective populations of inhabitants were evaluated separately, the same trend was observed (Figures 1B and 1C). Among the macroregions, the Northeast had the highest absolute number of hospitalizations, followed by the Southeast, South, North, and Central-West (Figure 1A). When analyzed by the number of inhabitants, the Northeast still had the highest hospitalization rate, followed by the North, South, Central-West, and Southeast (Figure 1D). When analyzed by the number of inhabitants and sex, the Northeast still had the highest hospitalization rate, followed by the North, South, Central-West, and Southeast (Figures 1E and F), according to sex.

**Figure 2:**
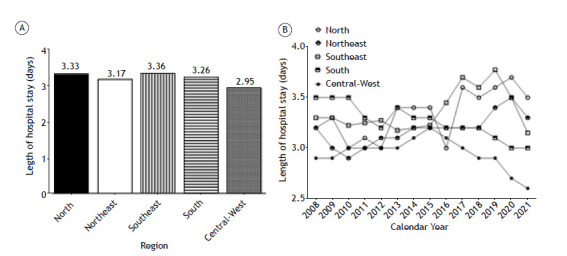
Mean length of hospital stay (in days) due to asthma and status asthmaticus by region throughout the study period (in A) and per year (in B).

To understand the characteristics of the hospitalization profile according to the macroregions, we calculated the mean number of hospitalization days from 2008 to 2021 and per year (Figure 2). Interestingly, the Southeast region had the greatest number of days per hospitalization, followed by the North, South, Northeast, and Central-West (Figure 2A). However, all regions exhibited high variability over the years (Figure 2B).

**Figure 3:**
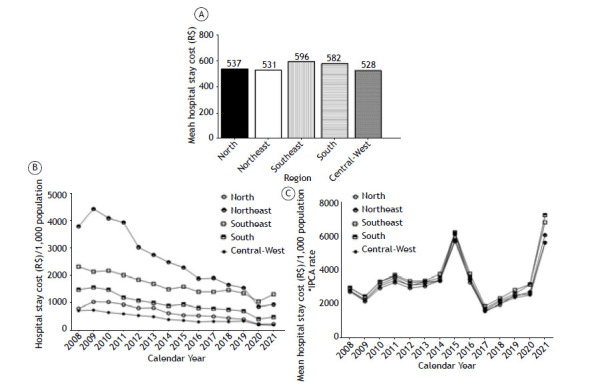
Mean hospital stay costs per region (in A). Total hospital stay costs by region per 1,000 population per year (in B). Mean hospital stay costs by region per 1,000 population normalized by monetary correction per year (in C). R$: Brazilian Real. *IPCA rate: monetary correction.

To analyze whether the length of hospital stay was associated with hospital expenditure, we analyzed the costs of hospital stay. The Southeast region spent a greater amount of financial resources, followed by the South, North, Northeast, and Central-West regions. From 2008 to 2021, financial resources decreased in all regions. When corrected by the IPCA, which represents the Brazilian inflation-targeting system, spending in all regions overlapped with peaks in 2015 and 2021 (Figure 3).

**Figure 4:**
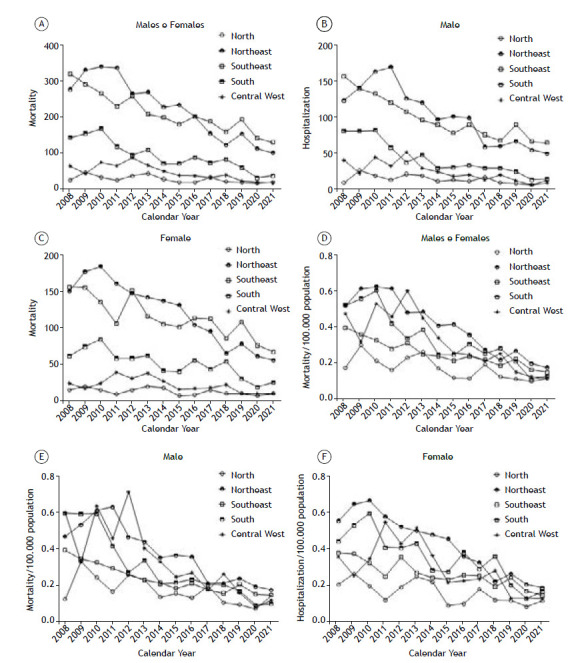
Asthma mortality per year by Brazilian region. In A, mortality in total population. In B, mortality in the male population. In C, mortality in the female population. In D, mortality per 100,000 population. In E, mortality per 100,000 males. In F, mortality per 100,000 females.

Regarding hospitalizations, the mortality rate also declined over the years. When analyzed by absolute numbers, the Southeast region presented the highest mortality, followed by the Northeast, South, North, and Central-West when both sexes were analyzed (Figure 4A). When the respective populations were evaluated separately, the same trend of reduction in mortality was observed (Figures 4B and 4C). When normalized, the proportional mortality rates also differed from the hospitalization rates, with the Northeast region having the highest proportion of deaths, followed by the Southeast, Central-West, and North regions (Figure 4D). When the data were normalized by population separately, we observed a reduction in mortality for both sexes in different regions (Figures 4E and F).

## DISCUSSION

The total number of hospitalizations and deaths due to asthma significantly decreased from 2008 to 2021. In the last year analyzed, 327 people died of asthma, approximately one death/day, and there were approximately 55,000 hospitalizations in Brazil. In the last thirteen years, there were reductions of 73% and 60% in the absolute number of hospitalizations and deaths due to asthma, respectively. The mean length of hospital stay for patients with asthma was approximately 3 days, irrespective of the region in Brazil. The costs of hospital admissions for asthma in Brazil decreased during the study period despite economic inflation or political instability.

In 2021, the last year analyzed, we observed approximately 1 death/day. Previous studies reported up to 5 deaths/day^12^ ^,^ ^19^ related to asthma in Brazil in the 2000s; however, they considered the total number of deaths related to the disease, such as people who may have died at home and/or who had died from other associated factors. On the other hand, our study reported deaths among hospitalized patients specifically due to asthma. Although the comparison is difficult, we can consider that the number of deaths reported in our study are more precise since the information presented can have fewer mistakes in determining the cause of death than those previously reported.^12^ ^,^ ^19^ ^)^ A study carried out by Cardoso et al.^10^ reported that, in 2013, 2,047 people died from asthma in Brazil (approximately 5 deaths/day), which was a greater number than that in our study. This is an important result for the Brazilian UHCS and can be explained by the use of pharmacological therapy for asthma (beclomethasone and albuterol), which was made available throughout the country free of charge by the Brazilian National Ministry of Health in 2009.^10^

Our results strongly suggest that asthma management in Brazil has improved over the years, as demonstrated by the reduction in hospitalizations and mortality. Decreases in hospitalization rates in the last two decades have been reported in other countries, such as Canada,^20^ Costa Rica,^21^ and Kuwait.^22^ This may be related to advances in pharmacological (e.g., immunobiologics) and nonpharmacological (e.g., education, exercise, and physical activity) treatments,^21^ as well as to the advancement of public policies aimed at this population. Tavakoli et al.^23^ observed a major increase in the use of ICSs in combination with LABAs starting in 2002 in the Canadian population, which coincided with the decline in hospital admission rates.^20^ Similarly, our study showed a substantial decrease in hospitalizations coinciding with the implementation of pharmacological therapy for asthma free of charge, made available throughout the country starting in 2011.^24^

The medication provided by the UHCS was previously shown to be cost-effective for asthma management.^25^ Even though there was a significant increase in asthma-related costs when ICS therapy was included in the “Popular Pharmacy Program” in 2011, our study showed a decrease in values in subsequent years, suggesting that improved pharmacotherapy was able to reduce other costs related to the disease. Another important aspect observed in our study was the significant decrease in hospitalizations and mortality from 2020 on, which may have been associated with the repercussions of COVID-19 and the measures employed by the WHO, such as stay-at-home orders.^26^ Our results corroborate the findings in the literature, in which there was a 36% reduction in hospitalizations in Scotland and Wales.^27^ Additionally, Shah et al.^28^ observed a significant reduction in primary care attendance for asthma exacerbations during the pandemic.

Indeed, at the beginning of the pandemic, there was a fear that SARS-CoV-2 could contribute to asthma exacerbations similarly to those caused by other respiratory viruses^29^; however, a meta-analysis suggested that individuals with asthma have a reduced risk of contracting COVID-19 when compared with those without the disease.^30^ Shah et al. also reported a significant reduction in the use of primary care for asthma exacerbations during the pandemic, likely due to social distancing measures and the increase in mask usage worldwide.^28^

We found that asthma hospitalization costs showed reduction differences among the Brazilian regions. The Southeast region had the highest economic expenditure, approximately R$ 596 (Brazilian real) or USD 119 per hospitalization. However, despite the decrease in asthma hospitalizations, the cost balance was still high when all Brazilian regions were included. Approximately R$ 2,776.50 were spent on hospital admissions for asthma between 2008 and 2021.

Indeed, spending on hospitalizations and medication may be the main cause of health system costs in Brazil related to COPD and severe asthma.^31^ ^,^ ^32^ Asthma contributes to high costs at the individual level that are directly associated with disease management and indirectly associated with social factors (impaired quality of life, absenteeism from school/work, and mental health impairment).^31^ A systematic review^32^ found that the cost from the perspective of the UHCS in Brazil, derived from two studies,^33^ ^,^ ^34^ revealed mean annual hospital costs per patient of USD 135 and USD 733, respectively. A study performed in the USA between 2008 and 2013 showed that the total annual cost of asthma, including medical care, absenteeism, and mortality, was USD 81.9 billion.^35^ In addition, the annual per-person medical cost of asthma was approximately USD 3,266. From the total budget, USD 1,830 were spent for prescriptions, USD 640 were spent for consultations, USD 529 were spent for hospitalizations, USD 176 were spent for outpatient visits, and USD 105 were spent for emergency care in the USA.

Our results are important for updating the asthma situation in Brazil. Despite the decrease in hospitalizations and deaths from asthma in Brazil, it is extremely important that new policies be employed to reduce both variables, for example, by improving education about disease management and physical activity in daily life, which has been demonstrated to contribute to better asthma control in this population.^36^ ^,^ ^37^

Our study has several limitations. The data analyzed were collected from electronic records. Although notification is mandatory, there is potential for missing data and inclusion of incorrect records, which may lead to underreporting of the disease. Another important aspect is the reduction in the number of hospitalizations from 2020 to 2021, which can be associated with the restriction measures recommended by the WHO due to COVID-19.

Our results showed that the number of asthma deaths and hospitalizations has decreased in Brazil in the last fifteen years. Although there is still much to be done regarding asthma, these data suggest that national asthma has improved in Brazil.

## References

[1] GBD Chronic Respiratory Disease Collaborators (2020). Prevalence and attributable health burden of chronic respiratory diseases, 1990-2017: a systematic analysis for the Global Burden of Disease Study 2017. Lancet Respir Med.

[2] Sociedade Brasileira de Pneumologia e Tisiologia (SBPT) (c2023). https://sbpt.org.br/portal/espaco-saude-respiratoria-asma.

[3] Forno E, Brandenburg DD, Castro-Rodriguez JA, Celis-Preciado CA, Holguin F, Licskai C (2022). Asthma in the Americas An Update: A Joint Perspective from the Brazilian Thoracic Society, Canadian Thoracic Society, Latin American Thoracic Society, and American Thoracic Society. Ann Am Thorac Soc.

[4] Hekking PW, Wener RR, Amelink M, Zwinderman AH, Bouvy ML, Bel EH (2015). The prevalence of severe refractory asthma. J Allergy Clin Immunol.

[5] Wang E, Wechsler ME, Tran TN, Heaney LG, Jones RC, Menzies-Gow AN (2021). Characterization of Severe Asthma Worldwide Data From the International Severe Asthma Registry [published correction appears in. Chest.

[6] Chung KF, Wenzel SE, Brozek JL, Bush A, Castro M, Sterk PJ (2014). International ERS/ATS guidelines on definition, evaluation and treatment of severe asthma [published correction appears in Eur Respir.

[7] Pizzichini MMM, Carvalho-Pinto RM, Cançado JED, Rubin AS, Cerci A, Cardoso AP (2020). 2020 Brazilian Thoracic Association recommendations for the management of asthma. J Bras Pneumol.

[8] Brasil. Ministério da Saúde (c2023). 71% dos brasileiros têm os serviços públicos de saúde como referência [about 2 screens].

[9] Ponte EV, Cruz AA, Athanazio R, Carvalho-Pinto R, Fernandes FLA, Barreto ML (2018). Urbanization is associated with increased asthma morbidity and mortality in Brazil. Clin Respir J.

[10] Cardoso TA, Roncada C, Silva ERD, Pinto LA, Jones MH, Stein RT (2017). The impact of asthma in Brazil a longitudinal analysis of data from a Brazilian national database system. J Bras Pneumol.

[11] World Health Organization. Chronic Respiratory Diseases and Arthritis. Management of Noncommunicable Diseases Department (c2023). Implementation of the WHO Strategy for Prevention and Control of Chronic Respiratory Diseases.

[12] Brito TS, Luiz RR, Silva JRLE, Campos HDS (2012). Asthma mortality in. Brazil, 1980-.

[13] Rodrigues MA, Facchini LA, Piccini RX, Tomasi E, Thumé E, Silveira DS (2008). Use of outpatient services by the elderly in the South and Northeast of Brazil [Article in Portuguese] Cad Saude. Publica.

[14] Viacava F, Bellido JG (2017). Health, access to services and sources of payment, according to household surveys [published correction appears in Cien Saude. Colet.

[15] Global Initiative for Asthma (GINA) (c2022). Global Strategy for Asthma Management and Prevention (2022 update).

[16] Brasil. Ministério da Saúde (2023). Tecnologia da Informação a Serviço do SUS (DATASUS) [homepage on the Internet].

[17] Instituto Brasileiro de Geografia e Estatística Censo 2010.

[18] Afonso PP, Afonso ML, Pinheiro DH, Collaço RC, Justo AF (2021). Decline in schizophrenia and schizophrenia spectrum disorders in Brazil a cross-sectional study from 2013 to 2019. Braz J Surg Clin Res.

[19] Souza-Machado C, Souza-Machado A, Coelho ACC, Amaral MTR, Cruz A (2012). Global Asthma Epidemiology 80 Asthma Mortality in Brazil (1998-2006). World Allergy Organ J.

[20] Lee TY, Petkau J, Mangat N, Safari A, Cragg JJ, Lynd LD (2022). 16-year trends in asthma hospital admissions in Canada. Ann Allergy Asthma Immunol.

[21] Soto-Martínez M, Avila L, Soto N, Chaves A, Celedón JC, Soto-Quiros ME (2014). Trends in hospitalizations and mortality from asthma in Costa Rica over a 12- to 15-year period. J Allergy Clin Immunol Pract.

[22] Ziyab AH, Abul AT (2014). Trends in asthma hospital admissions and mortality in. Kuwait, 2000-.

[23] Tavakoli H, Mark FitzGerald J, Lynd LD, Sadatsafavi M (2018). Predictors of inappropriate and excessive use of reliever medications in asthma a 16-year population-based study. BMC Pulm Med.

[24] Almeida ATC, Sá EB, Vieira FS, Benevides RPSE (2019). Impacts of a Brazilian pharmaceutical program on the health of chronic patients. Rev Saude Publica.

[25] Nair P (2011). Early interventions with inhaled corticosteroids in asthma benefits and risks. Curr Opin Pulm Med.

[26] World Health Organization (c2019). How to Protect yourself.

[27] Davies GA, Alsallakh MA, Sivakumaran S, Vasileiou E, Lyons RA, Robertson C (2021). Impact of COVID-19 lockdown on emergency asthma admissions and deaths national interrupted time series analyses for Scotland and Wales. Thorax.

[28] Shah SA, Quint JK, Nwaru BI, Sheikh A (2023). Impact of COVID-19 national lockdown on asthma exacerbations interrupted time-series analysis of English primary care data [published correction appears in. Thorax.

[29] Primary Care Respiratory Society (PCRS) (c2020). PCRS Pragmatic Guidance. Diagnosing and managing asthma attacks and people with COPD presenting in crisis during the UK Covid 19 epidemic.

[30] Sunjaya AP, Allida SM, Di Tanna GL, Jenkins C (2022). Asthma and risk of infection, hospitalization, ICU admission and mortality from COVID-19 Systematic review and meta-analysis. J Asthma.

[31] Campos Hda S, Lemos AC (2009). Asthma and COPD according to the pulmonologist. J Bras Pneumol.

[32] Roncada C, de Oliveira SG, Cidade SF, Sarria EE, Mattiello R, Ojeda BS (2016). Burden of asthma among inner-city children from Southern Brazil. J Asthma.

[33] Franco R, Santos AC, do Nascimento HF, Souza-Machado C, Ponte E, Souza-Machado A (2007). Cost-effectiveness analysis of a state funded programme for control of severe asthma. BMC Public Health.

[34] Santos LA, Oliveira MA, Faresin SM, Santoro IL, Fernandes AL (2007). Direct costs of asthma in Brazil a comparison between controlled and uncontrolled asthmatic patients. Braz J Med Biol Res.

[35] Nurmagambetov T, Kuwahara R, Garbe P (2018). The Economic Burden of Asthma in the United States, 2008-2013. Ann Am Thorac Soc.

[36] Freitas PD, Passos NFP, Carvalho-Pinto RM, Martins MA, Cavalheri V, Hill K (2021). A Behavior Change Intervention Aimed at Increasing Physical Activity Improves Clinical Control in Adults With Asthma A Randomized Controlled Trial. Chest.

[37] Passos NF, Freitas PD, Carvalho-Pinto RM, Cukier A, Carvalho CRF (2023). Increased physical activity reduces sleep disturbances in asthma A randomized controlled trial. Respirology.

